# Disomy 21 in spermatozoa and the paternal origin of trisomy 21 Down syndrome

**DOI:** 10.1186/s13039-015-0155-2

**Published:** 2015-08-19

**Authors:** Erik Iwarsson, Ulrik Kvist, Maj A. Hultén

**Affiliations:** Department of Molecular Medicine and Surgery, Karolinska Institutet, Karolinska University Hospital, S-171 76 Stockholm, Sweden; Department of Clinical Genetics, Karolinska University Hospital, S-171 76 Stockholm, Sweden; Centre for Andrology and Sexual Medicine, Karolinska University Hospital Huddinge, S-141 86 Stockholm, Sweden

**Keywords:** Trisomy 21, Down syndrome, Spermatozoa, Chromosome copy number, Fluorescence in situ hybridisation (FISH), Disomy 21, Paternal origin

## Abstract

**Background:**

Trisomy 21 Down syndrome is the most common genetic cause for congenital malformations and intellectual disability. It is well known that in the outstanding majority of cases the extra chromosome 21 originates from the mother but only in less than 10 % from the father. The mechanism underlying this striking difference in parental origin of Trisomy 21 Down syndrome is still unknown. However, it seems likely that the main reason is a much higher stringency in the elimination of any trisomy 21 cells during fetal testicular than ovarian development. We have here focussed attention on the paternal gametic output, i.e. the incidence of disomy 21 in spermatozoa.

**Results:**

We have used fluorescence in situ hybridisation (FISH) to determine the copy number of chromosome 21 in spermatozoa from 11 men with normal spermiograms. Due to the well-known risk of false positive and false negative signals using a single FISH probe, we have applied two chromosome 21q probes, and we have added a chromosome 18-specific probe to allow differentiation between disomy 21 and diploidy. Analysing a total number of 2000 spermatozoa per case, we documented an average incidence of disomy 21 at 0.13 %, with a range of 0.00-0.25 % and a SD of 0.08. There was no indication of diploidy in this cohort of 22,000 sperm.

**Conclusion:**

Numerous previous studies on the incidence of disomy 21 in sperm have been published, using FISH. As far as we are aware, none of these have applied more than a single chromosome 21-specific probe. Accepting our mean of 0.13 % of disomy 21, and providing there is no selective fertilisation capability of disomy 21 sperm in relation to the normal, we conclude that around 1 in 800 conceptions is expected to be trisomic for chromosome 21 of paternal origin. Bearing in mind that the maternal origin likely is at least 10 times more common, we tentatively propose that around 1 in 80 oocytes in the maternal ovarian reserve may be disomy 21. One reason for this discrepancy may be a more stringent selection against aberrant chromosome numbers during spermatogenesis than oogenesis. Further work is required to determine the relevant stages of spermatogenesis at which such a selection may take place.

## Background

Down syndrome is by far the most common genetic reason for congenital malformations and intellectual disability in the human population. In a recent survey of 29,256 cases, ascertained during 1989 to 2009 in the UK, where the diagnosis had been made by the Cytogenetic Laboratories, nearly 97 % of all cases were free trisomy 21. The incidence of live births in the whole cohort of cases was 1 per 1000 [1].

It is well known that the outstanding majority of cases with a free extra chromosome 21 originate from the mother, i.e. in over 90 % of cases, and less than 10 % is paternal. Naturally, much attention has focussed on the mechanism underlying the maternal origin, in particular the reason for the so-called maternal age effect, i.e. a drastic increase in incidence at advanced maternal age. It is now generally accepted that the majority of cases are due to a disturbance occurring during the long meiotic prophase (lasting from mid fetal life until menopause), leading to so-called primary non-disjunction of the two chromosomes 21 at the first meiotic division, taking place just before ovulation (see e.g. Table one in [2], [3–5]). In sharp contrast, we have concluded that the majority, if not all cases of T21, having an extra free chromosome 21, are not caused by any problem in the first meiotic cell division *per se*. Instead we suggest that the crucial factor is the general occurrence of pre-meiotic/mitotic T21 mosaicism, leading to so-called secondary meiotic non-disjunction of the oocytes having three chromosomes 21 [2, 6, 7]. This hypothesis, based on the occurrence of around 1/200 oogonia showing T21, has been criticised by Rowsey et al [5], who, using fluorescence in situ hybridisation (FISH) with a 13/21 centromere probe, could not detect a single T21 oocyte. They scored a total of 1,206 oocytes at the first meiotic/Leptotene, stage from seven fetal ovarian samples, i.e. less than 200 cells per case. It goes without saying that further work is required to either dismiss or confirm our hypothesis that pre-meiotic T21 mosaicism plays an outstanding role in the maternal origin of T21 Down syndrome.

By comparison, very little work has been devoted to the understanding of the paternal origin of T21 Down syndrome. We have previously investigated the incidence of T21 in fetal testicular cell nuclei from four male foetuses, following termination of pregnancy for a non-medical/social reason at gestational age 14-19 weeks. Using FISH with two chromosome–specific probes, and analysing at least 2000 premeiotic/mitotic cells per case, we could not detect a single cell with T21. We tentatively concluded that there is a much more stringent selection against aneuploidy during fetal spermatogenesis than oogenesis [8]. There is also an indication that a similar selection against aneuploidy may take place postmeiotically during spermiogenesis ([9], review in [10]).

On the other hand, a large number of studies have been devoted to the analysis of the incidence of disomy 21 in sperm obtained from normal controls. However, as far as we are aware, all of these studies have used a single chromosome 21 probe, and bearing in mind the possibility of false negatives, the raw values obtained have been multiplied by 2 [10]. We here present the results of scoring disomy 21 in sperm, obtained from 11 men with normal spermiograms. In order to avoid both false positives and false negatives [11, 12] we have in this study used the same two 21q-specific probes as in our previous investigations of the fetal testicular and ovarian fetal samples (Vysis 21q and Cytocell 21tel) together with a control chromosome 18 probe to allow differentiation between disomy 21 and diploidy (or disomy 21 plus disomy 18).

## Results and discussion

**Fig. 1:**
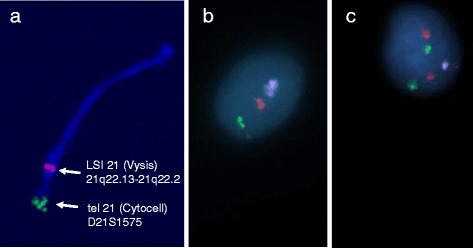
Examples of fluorescence in situ hybridisation (FISH) results, using two chromosome 21 specific probes (Vysis LSI 21 in red and Cytocell tel 21 in green). **a** Location of the probes near the end of the long arm of chromosome 21. Reproduced from [7] **b** A normal spermatozoon showing one dual chromosome 21-specific signal in combination with one chromosome 18 control probe signal (Vysis CEP18 in pink). **c** A spermatozoon showing two dual chromosome 21-specific signals (red and green) together with one chromosome 18 signal, therefore recorded as disomy 21.

The average incidence of chromosome 21 disomy in the sperm samples from this cohort of 11 men was 0.13 %, with a range of 0.00 %-0.25 %, SD 0.08 (Table 1). On this score, and provided there is no difference in fertilisation capability between the normal and the disomy 21 sperm, we may therefore expect around 1 per 800 conceptions to be trisomic for chromosome 21 of paternal origin. To our knowledge this is the first FISH study using two rather than a single probe for the estimation of the copy number of chromosome 21, an approach that we used for the analysis of 2000 spermatozoa per case. Examples of the FISH images in a normal monosomy 21 in comparison to a disomy 21 sperm head are shown in Fig. 1.

**Table 1:** Analysis of semen samples using FISH with two DNA-probes from chromosome 21, Vysis 21q22 (red) and Cytocell 21qtel (green) together with a control chromosome 18 probe, Abbot (blue), to allow differentiation between disomy and diploidy (or disomy 21 plus disomy 18)

No of signals from the two chr 21 DNA probes		1green/1red	2green/2red	2green/1red^1^	1green/2red^1^	1green/0red^1^	0green/1red^1^	Total no of cells
		(normal)	(disomy)					
Sample and age of the men	HP1	1988 (99.4 %)	5 (0.25 %)	3 (0.15 %)	4 (0.2 %)	0 (0.00 %)	0 (0.00 %)	2000
	32 y							
	HP2	1994 (99.7 %)	3 (0.15 %)	0 (0.00 %)	3 (0.15 %)	0 (0.00 %)	0 (0.00 %)	2000
	37 y							
	HP3	1992 (99.6 %)	2 (0.10 %)	2 (0.10 %)	4 (0.2 %)	0 (0.00 %)	0 (0.00 %)	2000
	39 y							
	HP4	1991 (99.55 %)	1 (0.05 %)	4 (0.2 %)	4 (0.2 %)	0 (0.00 %)	0 (0.00 %)	2000
	33 y							
	HP5	1978 (98.9 %)	0 (0.00 %)	3 (0.15 %)	14 (0.7 %)	5 (0.25 %)	0 (0.00 %)	2000
	45 y							
	3486	1991 (99.55 %)	3 (0.15 %)	4 (0.2 %)	2 (0.10 %)	0 (0.00 %)	0 (0.00 %)	2000
	28 y							
	3499	1993 (99.65 %)	4 (0.2 %)	2 (0.10 %)	1 (0.05 %)	0 (0.00 %)	0 (0.00 %)	2000
	53 y							
	3500	1992 (99.6 %)	2 (0.10 %)	2 (0.10 %)	3 (0.15 %)	0 (0.00 %)	1 (0.05 %)	2000
	30 y							
	3501	1986 (99.3 %)	2 (0.10 %)	1 (0.05 %)	9 (0.45 %)	0 (0.00 %)	2 (0.10 %)	2000
	42 y							
	3502	1992 (99.6 %)	5 (0.25 %)	1 (0.05 %)	1 (0.05 %)	1 (0.05 %)	0 (0.00 %)	2000
	40 y							
	3505	1995 (99.75 %)	1 (0.05 %)	0 (0.00 %)	3 (0.15 %)	1 (0.05 %)	0 (0.00 %)	2000
	35							
Mean		1990 (99.51 %)	2.5 (0.13 %)	2.0 (0.10 %)	4.4 (0.22 %)	0.6 (0.03 %)	0.3 (0.01 %)	
SD		4.5 (0.23 %)	1.6 (0.08 %)	1.3 (0.07 %)	3.7 (0.18 %)	1.4 (0.07 %)	0.6 (0.03 %)	

^1^Those spermatozoa showing either 2 green/1 red, 1 green/2 red, 1 green/0 red or 0 green/1 red signals were interpreted as false positives or false negatives, respectively, and were therefore excluded in the results of true disomy 21

Standard deviation (SD)

There are numerous investigations recording the rate of disomy 21 in sperm from apparently normal controls, using a single chromosome 21 probe. Results vary substantially in estimates of disomy 21 in individual sperm samples from 0.03-0.37 % [10]. To date it is not known to what extent this large variation reflects true biological variation or maybe due to technological problems in the FISH analysis of chromosome copy number, including in particular the use of a single chromosome 21-specific probe [13].

Our results, using two chromosome 21- specific probes, indicate that the average is in the order of 0.13 %. Interestingly, there are indications that there is no selection against chromosomally abnormal sperm at fertilization [14, 15].

By comparison to the large number of studies devoted to the study of sperm chromosome aneuploidy, there are so far only a few papers dealing with its mechanism of origin [8, 16, 14, 10]. The occurrence of disomy 21 in a sperm sample may in principle be due to mitotic mal-segregation at prenatal and postnatal development, as well as by first and second meiotic non-disjunction post puberty.

To our knowledge there are no adequate counts of chromosome 21 copy number in pre-meiotic, spermatogonial, metaphases from testicular biopsies of adult men. The information as regards the post-pubertal meiotic metaphase stages, Metaphase I (MI) and Metaphase II (MII) is also very limited. No chromosome 21 copy number changes were found by Uroz et al. [17] or Uroz and Templado [9] in a total sample of 485 spermatocytes at the MI stage from five normally fertile men. Likewise, neither Laurie et al. [18] nor Uroz et al. [17] identified any chromosome 21 copy number changes in a total of 266 cells at the MII stage from eight men. On the other hand, analysing 248 MII cells Uroz and Templado [9] detected one extra chromosome 21 in one MII cell, as well as one extra 21 chromatid in two out of the from three men (1.2 %).

In spite of the lack of a statistically significant paternal age effect [19, 20] it may then be prudent to pay special attention to the possibility of positive proliferative selection, leading to clustering of aberrant cell nuclei [21, 22]. Finally, it would be of added interest to compare the incidence of chromosome 21 copy number changes at the different stages of spermatogenesis with that in sperm as well as that in blood samples from the same men (see previous examples in Table two in [8], [23–25]). These preliminary data indicate that a combination of somatic and germinal chromosome 21 mosaicism may be common among men in the general population.

## Conclusion

Based on the results of this, the one and only evaluation of the incidence of disomy 21 in spermatozoa from men with normal spermiograms, using two differently coloured FISH probes, we conclude that in the order of 1 in 800 conceptions is expected to be trisomic for chromosome 21 of paternal origin. Bearing in mind the 10 times higher likelihood of maternal origin, we further suggest that the incidence of disomy 21 in oocytes, comprising the maternal ovarian reserve, may be in the order of 1 in 80. The mechanism(s) underlying this striking difference is not known but there are indications from the few studies so far performed that there is a more stringent selection against aneuploidy during spermatogenesis than during oogenesis.

## Methods

Semen samples were obtained from 11 healthy males with normal spermiograms, where the couples had been referred for investigation of infertility, and it had turned out that the female partners had been diagnosed as infertile. The samples were anonymised and all procedures performed with informed consent and ethical approval from the Regional Ethical Committee at Karolinska Institutet, reference number 03-010.

### Semen preparation

Spermatozoa from whole semen (n = 11) were washed three times in phosphate-buffered saline (PBS) at pH7.2. After an initial centrifugation of 1 ml of whole semen at 650 x g for 10 min, the supernatants were withdrawn and the sperm pellets were re-suspended in 2 mL of PBS. The procedure was repeated twice, and the final pellets were re-suspended and fixed in methanol/acetic acid (3:1). The samples were stored at -20 °C until used.

### FISH

Microscopy slides for FISH analysis were prepared by dripping 5 μl of spermatozoa suspension on clean slides, followed by air drying at room temperature. The slides were then washed in 2X standard saline citrate (SSC) and decondensed, using incubation in 4 mM dithiothreitol (DTT) for 5 min at room temperature. After additional washing in phosphate-buffered saline (PBS) and dehydration through a series of alcohol, the slides were left to air-dry at room temperature. Slides were then denatured in 70 % formamide at 73 °C for 2 min and dehydrated in an ethanol series at -20 °C.

Two DNA probes, positioned near the end of the long arm of chromosome 21 and labelled in SpectrumOrange and SpectrumGreen, respectively, were used (Vysis LSI 21 SpectrumOrange, Cat No: 05 J13-002, Abbot Molecular Inc, USA and Cytocell, Cat No. LPT21QG/R, Cytocell Technologies Ltd. UK). A chromosome 18 centromeric probe, labelled in SpectrumAqua, was added to be able to differentiate between disomy and diploidy (CEP 18 SpectrumAqua Probe (D18Z1), Cat No: 32-131018, Abbot Molecular Inc, USA). The DNA probes were mixed, denatured for 5 min at 73 °C and added to the slides, followed by hybridisation and post-hybridisation washing, according to the manufacturers’ instructions. After dehydration, slides were mounted in glycerol, containing 2.3 % DABCO (1, 4-diazabicyclo-(2, 2, 2) octane) as antifade and DAPI (4, 6,-diamino-2-phenyl-indole) 0.5 mg/ml for nuclear counterstaining.

Fluorescent signals were analysed, using a Zeiss Axioskop 2 microscope equipped with a cooled CCD camera (CoolSnap; Photometrics Ltd, USA), controlled by a Power Macintosh computer. Grey scale images were captured, pseudo-coloured and merged, using the SmartCapture 2 software (Digital Scientific Ltd, UK).
